# Burnout, does the university entrance test failing attribute? A Bangladeshi exploratory study

**DOI:** 10.1371/journal.pone.0258100

**Published:** 2021-10-05

**Authors:** Mohammed A. Mamun, Mariam Binte Safiq, Ismail Hosen, Firoj al Mamun

**Affiliations:** 1 CHINTA Research Bangladesh, Savar, Dhaka, Bangladesh; 2 Department of Public Health and Informatics, Jahangirnagar University, Savar, Dhaka, Bangladesh; University of Southern Queensland, AUSTRALIA

## Abstract

Getting admission into a university is highly competitive globally and can be regarded as a mental stressor for some individuals. In Bangladesh, most of the universities allow students to repeat the test, whereas repeat test-taking students are supposed to suffer from psychological issues like burnout because of academic non-achievement in their first attempt. But there is no prior study assessing burnout to the population, which was investigated herein. A cross-sectional study was carried out among a total of 911 Bangladeshi university entrance test-taking students (i.e., 49.5% first-time test-takers). The survey measures included socio-demographics, admission-related issues, and mental health problems (depression, anxiety, and burnout). Of the mental health problems, depression, anxiety, and burnout were assessed by the Patient Health Questionnaire (PHQ-9), Generalized anxiety disorder (GAD-7), and Maslach Burnout Inventory—Student Survey (MBI–SS), respectively. In light of two-dimensional and three-dimensional cutoff schemes of the MBI—SS, about 43.7% and 17.3% of the test-taking students, respectively, were classified to be burnout in the total sample. Additionally, 48.1%, 71.4%, and 49.1% of the students in the total sample reported higher emotional exhaustion, higher cynicism, and lower academic efficacy, respectively. In respect to the student status, two-dimensional burnout (48.0% vs. 39.2%; χ^2^ = 7.164, *p* = 0.007), and emotional exhaustion (52.2% vs. 43.9%, χ^2^ = 6.789, *p* = 0.034) were significantly higher among the repeat test-taking students. Satisfaction with previous mock tests, depression, and anxiety were the associated factors of burnout in all three samples. Based on the present study, it can be concluded that the university entrance test appearing students are highly prevalent to burnout, and the rate increases with academic non-achievement. As the first study assessing burnout to the population, the findings are anticipated to be helpful for policymaking and further studies both inside and outside Bangladesh.

## 1 Introduction

Because of prolonged or repeated stress, emotional and mental exhaustion (along with physical exhaustion in some cases), burnout occurs [1]. Burnout, the term for the first-time is coined by Freudenberger [2] as ‘staff burnout’, which is described as ‘to fail, wear out, or become exhausted making excessive demands on energy, strength, or resources, and can still be seen in the modern definitions of job burnout’ [1]. Later on, Maslach and colleagues [3] attempt to apply the term more generally. There are three fundamental components of burnout, namely (i) emotional exhaustion, (ii) depersonalization or cynicism, and (ii) reduced professional efficacy [1]. The normal life cycle of being stressed and/or burnout can be noted when someone first starts to feel energy depletion or exhaustion, which leads to increased mental detachment from their jobs, or feelings of negativism or cynicism related to their job; and as a result, they have to experience reduced professional efficacy or achievement [1, 4]. Although many studies are carried out investigating the potential comorbid factors of burnout in aiming to recognize it as a clinical disorder, yet it is not recognized as a medical condition officially. The World Health Organization recently classifies it as an occupational phenomenon in their latest International Classification of Diseases (ICD-11) [4].

In 2019, a total of 1,336,629 high school students appeared in the Higher Secondary School Certificate (HSC) and equivalent exams, the prior public exam to the university entrance test, and 73.93% of them passed [5]. Of these successful students, approximately 3.54% achieved GPA-5 (Grade Point Average—5), the highest results in the test. However, the next step of academic life, undergraduate education in Bangladesh, starts with a mandatory prior entrance admission test. But, because of limited seats (i.e., around 50-thousand seats in public universities), the university authorities can allow enrollment of nearly 15% of the high school graduates who are eligible by merits for appearing in the tests [6, 7]. Thus, the university admission test in Bangladesh can be regarded as one of the most competitive exam for students. However, the students are allowed to appear in the exam two times by most universities [8]. Therefore, there is a large number of high school graduates repeat the test to enter the university. The repeat test-taking students may be under excessive stress because of being (i) prolonged wait for the next test, (ii) under regret for losing one academic year from their life, (iii) under inferiority of their luckless preparation, etc. Besides, social negligence from neighbors and relatives towards the repeat test takers may also be regarded as a contributing emotional factor. Thus, it is observed that the repeat test takers are psychologically more vulnerable than the fresher ones. For instance, depression and anxiety prevalence rates among the repeat test-takers are reported to be 51.2% and 32.2%, respectively, whereas it is 40.9% and 24.2%, for the first-timers, reported by a recent Bangladeshi study [8].

There is enough evidence on linking between burnout and other psychological distresses. For instance, Bianchi and colleagues [9] try to conclude that burnout and depressive symptoms are linked in a way that is impossible to disentangle or separate. Similar assertions can be made for anxiety and burnout [10]. In addition, a considerable portion of the Bangladeshi students’ suicide is being reported as of academic distress and failure, a situation that is very indistinguishable from students’ burnout and extreme level of psychological suffering. For instance, Mamun et al. [11] observe academic distress and its failure to be the second most suicide reason after relationship problems in the general students’ suicide (17.9% vs. 19.6%), which is the first reason for medical sciences students in Bangladesh [12]. Even though failing in the university admission test leads to suicide in Bangladesh and its neighboring country such as India [13, 14]. Commensurately with the indications, the repeat test-taking students can be anticipated to have higher burnout compared to the fresher test-takers, although there is no prior evidence on such claim from the context of Bangladesh. Hence, the present study, first-ever, attempts to investigate whether failing in the first university entrance test may increase burnout or not by considering two groups (i.e., first-time vs. repeat test-takers).

## 2 Methods

### 2.1 Study participants and procedure

The present cross-sectional study was carried out at the Jahangirnagar University, Savar, Dhaka, Bangladesh. The survey was conducted between September 22 to October 1, 2019, during the university entrance test. Note, this university allows students to appear in the entrance test twice a time. That means, if someone fails to pass in his/her first attempt, then the individual can try again to get admitted to this university. However, the eligible participants for the study were those who resided in the university dorms during the admission test period and were interested to participate in the study. Before initiating the data collection, a short session (about 10–15 minutes) was organized by the research team about filling up the questionnaire and their queries about the questionnaire. After that, the participants were asked to fill-up the questionnaire through a self-administered process. Approximately 935 students were surveyed, from where 911 data were used for final analysis after removing the incomplete questionnaire.

### 2.2 Measures

#### 2.2.1. Sociodemographic factors

The basic socio-demographics such as gender, permanent residence, religion, family income, etc., were asked in the study. For assessing monthly family income, the study followed the suggested scheme (i.e., less than 15,000 Bangladeshi Taka [BDT] = lower class, 15,000–30,000 BDT = middle class, and more than 30000 BDT = upper class) by the previous researchers [15, 16]. Participants smoking and drug use status were also asked in the survey.

#### 2.2.2. Admission-related variables

The participants were categorized into two groups (i.e., first-time test takers vs. repeat test-takers) based on their appearance in the university entrance test. The GPA was ranked as poor (<4.5 GPA), moderate (4.5–4.99 GPA), and high (5 GPA). For the entrance admission test, if any professional coaching centers guided students were also asked. In addition, their performance in the coaching tests, average monthly expenditure for study-related costs between the period of prior public examination and entrance test (i.e., less than 5,000 BDT, 5,000–10,000 BDT, and more than 10,000 BDT), desired institute for getting admission (i.e., general university, engineering university, medical college, and agriculture university), and their educational background before the pre-university test (i.e., science, commerce, or arts) related information were collected.

#### 2.2.3. Patient Health Questionnaire

Depression was assessed by the Patient Health Questionnaire (PHQ-9) [17]. The scale consists of a total 9-item based on a four-point Likert scale response (0 = not at all to 3 = nearly every day) considering 14 days before the data collection. The score ranges from 0 to 27, and a score of ≥10 was considered for donating depression cutoff score [18]. This cutoff score has a 88% sensitivity and 88% specificity for depression screening [17]. In the present study, Cronbach’s alpha was good (0.73).

#### 2.2.4. Generalized Anxiety Disorder

Anxiety was assessed by the Generalized Anxiety Disorder (GAD-7) [19]. The scale consists of a total 7-item based on a four-point Likert scale response (0 = not at all to 3 = nearly every day) considering 14 days before the data collection. The scores ranges from 0 to 21, and a score of ≥10 was considered for donating anxiety cutoff score [8]. This cutoff score has an 89% sensitivity and 82% specificity for screening anxiety [19]. In the present study, Cronbach’s alpha was good (0.84).

#### 2.2.5. Maslach Burnout Inventory—Student Survey

Academic burnout was assessed by the Maslach Burnout Inventory—Student Survey (MBI–SS) [20]. The MBI -SS comprises 15 items constituting three subscales–(i) exhaustion (consists of 4-item), (ii) cynicism (consists of 5-item), and (iii) efficacy (consists of 6-item). All items are scored on a 7-point Likert scale ranging from 1 (strongly disagree) to 7 (strongly agree). The scoring of the subscales is followed as emotional exhaustion (low = 0–9; moderate = 10–14; high > 14), cynicism (low = 0–1; moderate = 2–6; high > 6), and academic efficacy (low ≤ 22; moderate = 23–27; high ≥ 28) [21]. Higher scores on exhaustion and cynicism and lower scores on efficacy are the indicator for burnout. Previous studies have provided evidence for the scales’ reliability and its validity in university students [21, 22]. In the present study, Cronbach’s alpha values were 0.83 (emotional exhaustion), 0.74 (cynicism), and 0.80 (academic efficacy).

### 2.6. Ethical considerations

This study followed the medical research guidelines as suggested by the Helsinki Declaration. Besides, the ethics committee granted a formal IRB approval at the Institute of Allergy and Clinical Immunology of Bangladesh (Reference no: IRBIACIB/CEC/03201912/273). All of the participants was informed about the study purpose, voluntary participation, rights of withdrawing from the study during any phases, etc., and formal written consent was collected before their participation.

### 2.7. Statistical analysis

The data were analyzed using Statistical Package for Social Science (SPSS) Version 22.0 (SPSS Inc, Chicago, IL, USA). Microsoft Excel version 2019 was used for the data entry and cleaning. Descriptive statistics (i.e., percentage, frequencies) and inferential statistics (e.g., chi-square tests) were performed to see the relationships between the variables and outcome in the three samples. A binary logistic regression analysis was also performed in the study. There are two schemes for categorizing burnout; (i) two-dimensional criteria (high scores for both emotional exhaustion and cynicism) and (ii) three-dimensional criteria (high scores for both emotional exhaustion and cynicism and low scores for academic efficacy) [21, 23]. All inferential statistics were performed with two-dimensional burnout [21], considering the *p-*value <0.05 with a 95% confidence interval as significant.

## 3 Results

### 3.1 Characteristics of the participants

**Table 1** presents that majority of the sample were male (53.8%, n = 490; N = 911), belonged to a nuclear family (79.3%), were Muslim in religion (83.6%), and came from villages (69.7%). Among them, 7.6% were cigarette smokers, and 1.5% had a habit of taking drugs. Both first-timers (i.e., 49.5%, n = 451) and repeat test takers equally participated in the survey. Professional coaching centers coached three-fourths (77.2%) of the participants, and 87.7% wanted to get admitted into a university. About 68.1% were satisfied with their previous mock tests, and three-quarters (73.4%) had come from a science background. The overall depression prevalence rate was 47.9%, which was slightly higher among the repeat test-taking students (53.7% vs. 41.9%); and 28.9% was for anxiety in the total sample, which was lower in the first-time test takers (33.9% vs. 23.7%) (**Table 1**).

**Table 1:** Distribution of the variables among first-time and repeat university entrance test-takers.

Variables	Total (n; %)	First-time test-takers (*n;* %)	Repeat test-takers (*n;* %)	χ^2^ test value	df	*p*-value
**Sociodemographic variables**						
**Gender**						
Female	421 (46.2)	196 (43.5)	225 (48.9)	2.725	1	0.099
Male	490 (53.8)	255 (56.5)	235 (51.1)			
**Permanent residence**						
Rural	635 (69.7)	331 (73.9)	304 (66.5)	5.859	1	**0.015**
Urban	270 (29.6)	117 (26.1)	153 (33.5)			
**Religion**						
Muslim	762 (83.6)	367 (81.7)	395 (86.2)	3.430	1	0.064
Others	145 (15.9)	82 (18.3)	63 (13.8)			
**Family type**						
Nuclear	716 (79.3)	348 (78.0)	368 (80.5)	0.858	1	0.354
Joint	187 (20.7)	98 (22.0)	89 (19.5)			
**Monthly family income (BDT)**						
<15000	261 (35.0)	140 (38.5)	121 (31.7)	8.252	2	**0.016**
15000–3000	274 (36.7)	115 (31.6)	159 (41.6)			
>30000	211 (28.3)	109 (29.9)	102 (26.7)			
**Cigarette smoking status**						
Yes	69 (7.6)	31 (6.9)	38 (8.3)	0.596	1	0.440
No	840 (92.4)	418 (93.1)	422 (91.7)			
**Drug usage status**						
Yes	14 (1.5)	9 (2.0)	5 (1.1)	1.271	1	0.259
No	890 (98.5)	437 (98.0)	453 (98.9)			
**Admission-related variables**						
**Appearance in admission test**						
First time	451 (49.5)	-	-	-	-	-
Second time	460 (50.5)	-	-			
**Secondary School Certificate grade point average (SSC GPA)**						
Poor (<4.5)	160 (17.9)	85 (19.2)	75 (16.6)	11.049	2	**0.004**
Moderate (4.5–4.99)	272 (30.4)	153 (34.5)	119 (26.3)			
High (5)	464 (51.8)	205 (46.3)	259 (57.2)			
**Higher Secondary School Certificate grade point average (HSC GPA)**						
Poor (<4.5)	557 (62.4)	263 (59.5)	294 (65.2)	5.538	2	0.063
Moderate (4.5–4.99)	212 (23.7)	106 (24.0)	106 (23.5)			
High (5)	124 (13.9)	73 (16.5)	51 (11.3)			
**Coached by professional coaching centers**						
No	205 (22.8)	52 (11.7)	153 (33.6)	61.307	1	**<0.001**
Yes	694 (77.2)	392 (88.3)	302 (66.4)			
**Desired institute for admission**						
General university	786 (87.7)	380 (85.8)	406 (89.6)	8.451	3	**0.038**
Medical college	91 (10.2)	51 (11.5)	40 (8.8)			
Engineering university	14 (1.6)	11 (2.5)	3 (0.7)			
Agriculture university	5 (0.6)	1 (0.2)	4 (0.9)			
**Satisfied with previous mock tests**						
Yes	575 (68.1)	273 (63.6)	302 (72.8)	8.106	1	**0.004**
No	269 (31.9)	156 (36.4)	113 (27.2)			
**Average monthly expenditure (BDT)**						
<5,000	193 (24.2)	75 (19.5)	118 (28.6)	20.744	2	**<0.001**
5000–10,000	349 (43.7)	158 (41.0)	191 (46.2)			
>10,000	256 (32.1)	152 (39.5)	104 (25.2)			
**Educational background**						
Science	667 (73.4)	311 (69.1)	356 (77.6)	19.915	2	**<0.001**
Arts	184 (20.2)	117 (26.0)	67 (14.6)			
Commerce	58 (6.4)	22 (4.9)	36 (7.8)			
**Psychological suffering**						
**Depression**						
No	475 (52.1)	262 (58.1)	213 (46.3)	12.683	1	**<0.001**
Yes	436 (47.9)	189 (41.9)	247 (53.7)			
**Anxiety**						
No	648 (71.1)	344 (76.3)	304 (66.1)	11.511	1	**<0.001**
Yes	263 (28.9)	107 (23.7)	156 (3.9)			

### 3.2. Distribution of the burnout across the student status

**Fig 1:**
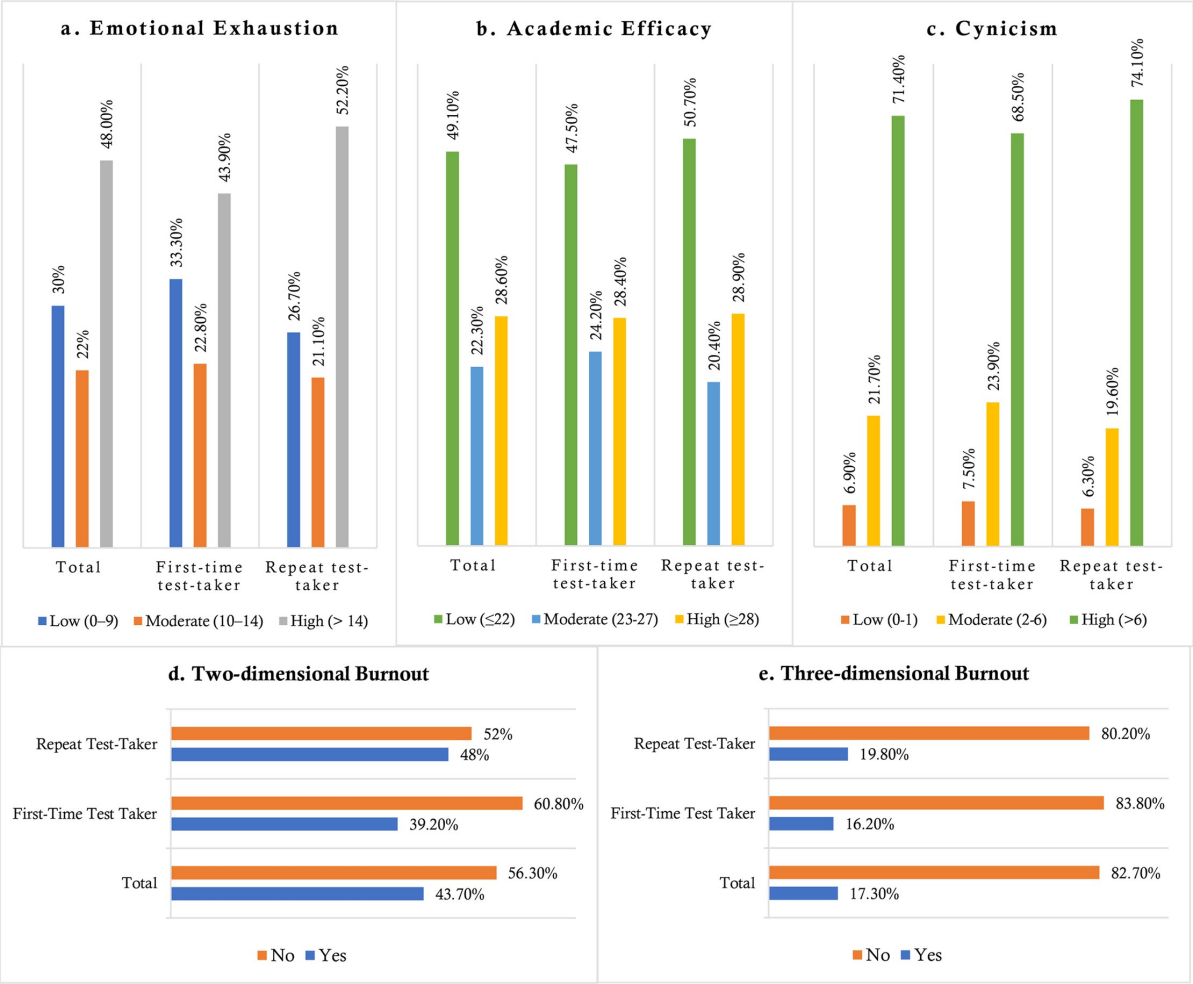
Student status-based distribution of (a) emotional exhaustion, (b) academic efficacy, (c) cynicism, (d) two-dimensional burnout, and (e) three-dimensional burnout.

**Fig 1** represents the distribution of burnout and its components across three groups. For instance, 48.1% and 71.4% of the students in the total sample were highly emotionally exhausted and cynic, whereas 49.1% reported having lower academic efficacy. The repeat test-taking participants belonged to have higher emotional exhaustion compared to the first-timers (i.e., 52.2% vs. 43.9%, χ^2^ = 6.789, df = 2, *p* = 0.034), although cynicism (for higher, 74.1% vs. 68.5%, χ^2^ = 3.520, df = 2, *p* = 0.172), and academic efficacy (for lower, 50.7% vs. 47.5%, χ^2^ = 1.923, df = 2, *p* = 0.382) were not significantly associated in terms of student status. About 43.7% (n = 398) and 17.3% (n = 158) of students in the total sample were classified as burnout based on two-dimensional and three-dimensional cutoff scores. Although, the repeat test takers were significantly prone to suffering from two-dimensional burnout (i.e., 48.0% vs. 39.2%; χ^2^ = 7.164, df = 1, *p* = 0.007), three-dimensional did not show any significant relationship across two student groups (χ^2^ = 1.995, df = 1, *p* = 0.158) (**Fig 1**).

### 3.3. Relationship between the studied variables and burnout

Gender was not significantly associated with burnout in the total sample and repeat test-takers, but it was associated with the first-time test-taking students (44.9% vs. 34.9%%; χ^2^ = 4.644, df = 1, *p* = 0.031 for female vs. male). Considering the residence, the participants from an urban area experienced more burnout syndrome in the total sample and first-time test takers. Smoker students belonging to the first-time test-taking group (i.e., 58.1% vs. 37.8%; χ^2^ = 4.973, df = 1, *p* = 0.026) and drug users belonging to the total sample (i.e., 71.4% vs. 43.3%; χ^2^ = 4.446, df = 1, *p* = 0.035) were reported to have higher burnout suffering. Similarly, the SSC GPA was associated with the total sample burnout, but the HSC GPA was associated with the total and first-timer sample. The students reporting satisfied with their mock test from the three groups suffered more burnout syndrome (50.8% vs. 27.9%; χ^2^ = 39.112, df = 1, *p*<0.001 for total sample; 45.4% vs. 28.2%; χ^2^ = 12.350, df = 1, *p*<0.001 for freshers; and 55.6% vs. 27.4%; χ^2^ = 26.193, df = 1, *p*<0.001 for repeaters). Lastly, participants experiencing psychiatric problems such as depression and anxiety were more vulnerable to burnout in all three samples (**Table 2**).

**Table 2:** Distribution of two-dimensional burnout among first-time and repeat university entrance test-takers.

Variables	Total (n; %)			First-time test-takers (*n;* %)			Repeat test-takers (*n;* %)		
	Yes (%)	*χ*^*2*^ test value (df)	*p*-value	Yes (%)	*χ*^*2*^ test value (df)	*p*-value	Yes (%)	*χ*^*2*^ test value (df)	*p*-value
**Sociodemographic variables**									
**Gender**									
Female	197 (46.8)	3.068 (1)	0.080	88 (44.9)	4.644 (1)	**0.031**	109 (48.4)	0.028 (1)	0.866
Male	201 (41.0)			89 (34.9)			112 (47.7)		
**Permanent residence**									
Rural	262 (41.3)	4.485 (1)	**0.034**	119 (36.0)	5.153 (1)	**0.023**	143 (47.0)	0.283 (1)	0.595
Urban	132 (48.9)			56 (47.9)			76 (49.7)		
**Religion**									
Muslim	334 (43.8)	0.154 (1)	0.695	148 (40.3)	1.074 (1)	0.300	186 (47.1)	0.610 (1)	0.435
Others	61 (42.1)			28 (34.1)			33 (52.4)		
**Family type**									
Nuclear	312 (43.6)	0.213 (1)	0.645	140 (40.2)	0.196 (1)	0.658	172 (46.7)	1.485 (1)	0.223
Joint	85 (45.5)			37 (37.8)			48 (53.9)		
**Monthly family income (BDT)**									
<15000	115 (44.1)	0.099 (2)	0.952	55 (39.3)	0.391 (2)	0.822	60 (49.6)	0.475 (2)	0.789
15000–3000	124 (45.3)			44 (38.3)			80 (50.3)		
>30000	93 (44.1)			46 (42.2)			47 (46.1)		
**Cigarette smoking status**									
Yes	35 (50.7)	1.509 (1)	0.219	18 (58.1)	4.973 (1)	**0.026**	17 (44.7)	0.181 (1)	0.670
No	362 (43.1)			158 (37.8)			204 (48.3)		
**Drug usage status**									
Yes	10 (71.4)	4.446 (1)	**0.035**	6 (66.7)	2.952 (1)	0.086	4 (80)	2.040 (1)	0.153
No	385 (43.3)			168 (38.4)			217 (47.9)		
**Admission-related variables**									
**Secondary School Certificate grade point average (SSC GPA)**									
Poor (<4.5)	67 (41.9)	6.731 (2)	**0.035**	31 (36.5)	2.439 (2)	0.295	36 (48.0)	3.441 (2)	0.179
Moderate (4.5–4.99)	102 (37.5)			54 (35.3)			48 (40.3)		
High (5)	219 (47.2)			88 (42.9)			131 (50.6)		
**Higher Secondary School Certificate grade point average (HSC GPA)**									
Poor (<4.5)	232 (41.7)	6.877 (2)	**0.032**	103 (39.2)	6.030 (2)	**0.049**	129 (43.9)	5.660 (2)	0.059
Moderate (4.5–4.99)	87 (41.0)			33 (31.1)			54 (50.9)		
High (5)	67 (54.0)			36 (49.3)			31 (60.8)		
**Coached by professional coaching centers**									
No	81 (39.5)	1.907 (1)	0.167	19 (36.5)	0.146 (1)	0.703	62 (40.5)	5.657 (1)	0.017
Yes	312 (45.0)			154 (39.3)			158 (52.3)		
**Desired institute for admission**									
General university	336 (42.7)	8.901 (3)	**0.031**	146 (38.4)	9.083 (3)	**0.028**	190 (46.8)	5.130 (3)	0.163
Medical college	45 (49.5)			20 (39.2)			25 (62.5)		
Engineering university	11 (78.6)			9 (81.8)			2 (66.7)		
Agriculture university	3 (60.0)			0 (0.0)			3 (75.0)		
**Satisfied with previous mock tests**									
Yes	292 (50.8)	39.112 (1)	**<0.001**	124 (45.4)	12.350 (1)	**<0.001**	168 (55.6)	26.193 (1)	**<0.001**
No	75 (27.9)			44 (28.2)			31 (27.4)		
**Average monthly expenditure (BDT)**									
<5,000	85 (44.0)	0.415 (2)	0.813	32 (42.7)	1.636 (2)	0.441	53 (44.9)	0.846 (2)	0.655
5000–10,000	149 (42.7)			56 (35.4)			93 (48.7)		
>10,000	116 (45.3)			63 (41.4)			53 (51.0)		
**Educational background**									
Science	305 (45.7)	5.209 (2)	0.074	125 (40.2)	2.439 (2)	0.295	180 (50.6)	4.566 (2)	0.102
Arts	67 (36.4)			40 (34.2)			27 (40.3)		
Commerce	24 (41.4)			11 (50.0)			13 (36.1)		
**Psychological suffering**									
**Depression**									
No	157 (33.1)	45.634 (1)	**<0.001**	83 (31.7)	15.013 (1)	**<0.001**	74 (34.7)	28.118 (1)	**<0.001**
Yes	241 (55.3)			94 (49.7)			147 (59.5)		
**Anxiety**									
No	241 (37.2)	38.511 (1)	**<0.001**	118 (34.3)	14.863 (1)	**<0.001**	123 (40.5)	20.649 (1)	**<0.001**
Yes	157 (59.7)			59 (55.1)			98 (62.8)		

### 3.4. Associated factors of the student burnout

**Table 3** represents the factors associated with two-dimensional burnout across three groups. In the total sample, living in urban areas, having poor or higher SSC and HSC GPA compared to moderate GPA, being satisfied with the previous mock test, being depressed, and being anxious were detected as the associated factors of burnout. Similarly, being female, living in an urban area, smoking, satisfaction with previous mock tests, depression, and anxiety were also significant predictors for burnout for the fresher test takers. Lastly, being coached by professional coaching centers, satisfaction with previous mock tests, suffering from depression, and anxiety were the burnout-associated factors for the repeat test takers (**Table 3**).

**Table 3:** Binary logistic regression analysis concerning two-dimensional burnout among first-time and repeat university entrance test-takers.

Variables	Total sample		First-time test-takers		Repeat test-takers	
	OR; 95% CI	*p*-value	OR; 95% CI	*p*-value	OR; 95% CI	*p*-value
**Sociodemographic variables**						
**Gender**						
Female	1.265 (0.972–1.645)	0.080	1.520 (1.038–2.226)	**0.032**	1.032 (0.716–1.488)	0.866
Male	Reference		Reference		Reference	
**Permanent residence**						
Rural	0.734 (0.552–0.978)	**0.034**	0.611 (0.399–0.937)	**0.024**	0.900 (0.610–1.328)	0.595
Urban	Reference		Reference		Reference	
**Religion**						
Muslim	1.075 (0.750–1.539)	0.695	1.303 (0.789–2.513)	0.301	0.809 (0.475–1.378)	0.435
Others	Reference		Reference		Reference	
**Family type**						
Nuclear	0.927 (0.671–1.281)	0.645	1.110 (0.700–1.760)	0.658	0.750 (0.471–1.193)	0.224
Joint	Reference		Reference		Reference	
**Monthly family income (BDT)**						
<15000	0.999 (0.693–1.440)	0.952	0.886 (0.532–1.475)	0.822	1.151 (0.679–1.951)	0.789
15000–3000	1.049 (0.731–1.505)		0.849 (0.497–1.449)		1.185 (0.720–1.950)	
>30000	Reference		Reference		Reference	
**Cigarette smoking status**						
Yes	1.359 (0.832–2.222)	0.221	2.278 (1.087–4.777)	**0.029**	0.865 (0.444–1.686)	0.670
No	Reference		Reference		Reference	
**Drug usage status**						
Yes	3.279 (1.021–10.535)	**0.046**	3.202 (0.790–12.976)	0.103	4.350 (0.482–39.223)	0.190
No	Reference		Reference		Reference	
**Admission-related variables**						
**Secondary School Certificate grade point average (SSC GPA)**						
Poor (<4.5)	0.806 (0.561–1.159)	**0.035**	0.763 (0.453–1.285)	0.296	0.902 (0.539–1.509)	0.181
Moderate (4.5–4.99)	0.671 (0.494–0.911)		0.725 (0.471–1.117)		0.661 (0.425–1.026)	
High (5)	Reference		Reference		Reference	
**Higher Secondary School Certificate grade point average (HSC GPA)**						
Poor (<4.5)	0.607 (0.411–0.898)	**0.034**	0.662 (0.393–1.114)	0.051	0.504 (0.275–0.926)	0.062
Moderate (4.5–4.99)	0.592 (0.379–0.926)		0.465 (0.251–0.860)		0.670 (0.340–1.321)	
High (5)	Reference		Reference		Reference	
**Coached by professional coaching centers**						
No	0.800 (0.582–1.099)	0.168	0.890 (0.488–1.621)	0.703	0.621 (0.419–0.921)	**0.018**
Yes	Reference		Reference		Reference	
**Desired institute for admission**						
General university	0.498 (0.083–2.996)	0.053	1008337740 (0.000-)	0.099	0.293 (0.30–2.843)	0.178
Medical college	0.652 (0.104–4.089)		1042647067 (0.000-)		0.556 (0.053–5.837)	
Engineering university	2.444 (0.271–22.016)		7272463291 (0.000-)		0.667 (0.025–18.059)	
Agriculture university	Reference		Reference		Reference	
**Satisfied with previous mock tests**						
Yes	2.669 (1.952–3.649)	**<0.001**	2.118 (1.389–3.232)	**<0.001**	3.316 (2.070–5.314)	**<0.001**
No	Reference		Reference		Reference	
**Average monthly expenditure (BDT)**						
<5,000	0.950 (0.652–1.383)	0.813	1.051 (0.601–1.840)	0.442	0.785 (0.462–1.331)	0.655
5000–10,000	0.899 (0.650–1.244)		0.776 (0.490–1.227)		0.913 (0.566–1.473)	
>10,000	Reference		Reference		Reference	
**Educational background**						
Science	1.194 (0.693–2.057)	0.075	0.672 (0.283–1.598)	0.299	1.809 (0.889–3.684)	0.105
Arts	0.811 (0.444–1.482)		0.519 (0.207–1.302)		1.194 (0.517–2.758)	
Commerce	Reference		Reference		Reference	
**Psychological suffering**						
**Depression**						
No	0.399 (0.305–0.523)	**<0.001**	0.469 (0.319–0.689)	**<0.001**	0.362 (0.248–0.529)	**<0.001**
Yes	Reference		Reference		Reference	
**Anxiety**						
No	0.400 (0.298–0.536)	**<0.001**	0.425 (0.273–0.660)	**<0.001**	0.402 (0.270–0.598)	**<0.001**
Yes	Reference		Reference		Reference	

OR, Odds Ratio; CI, Confidence Interval; BDT, Bangladeshi Taka.

## 4 Discussion

The overall sample reports that 43.7% and 17.3% of the university entrance test-taking students are burnout based on two-dimensional and three-dimensional cutoff schemes, respectively. This rate can be compared with a previous study on Brazilian medical students using a similar tool and scoring scheme [21]. The present study’s two-dimensional burnout is slightly lower than the Brazilian study (i.e., 44.9%), but a much higher rate is found for three-dimensional burnout (i.e., 26.4%). The prevalence of two-dimensional burnout is also reported to be 43.43% in Bahraini medical students [24], 35.9% in Brazilian medical internship students [25], 35.5% in Portugal medical students [26]; whereas using Copenhagen Burnout Inventory, 48.53% and 48.8% moderate to higher burnout is reported from Nepalese and Indian medical students, respectively [27, 28]. Correspondingly, 28.4% three-dimensional burnout is reported in Thailand [29], whereas it is 10.3% and 25.9% for Brazilian and Portugal samples, respectively [25, 26]. As the studies using different tools for assessing burnout with varying schemes of cutoff; thus, the comparison between findings from other studies is limited. However, it is well-established that burnout is highly prevalent in the healthcare professionals and students compared to any different populations because burnout is a state of mental and physical exhaustion related to work or caregiving activities that is more common in medical-related environments [30]. Thus, the present sample (i.e., university admission test takers) can be treated as a vulnerable cohort towards burnout after medical students or professionals.

In respect to the student status, the repeat test takers are more significantly prone to be emotionally exhausted. Because the repeat test-taking students fail to get admitted into university in their first attempt, which can be typically treated as academic non-achievement, a burnout factor [31]. Besides, the participants report differences in burnout rates on two-dimensional cutoff criteria across the student test appearing status, although no significant association is observed for three-dimensional burnout. This may be because of the time exposure difference of burnout factors (i.e., admission test occurrence) for the two groups. For instance, there is only a one-year difference between the two groups for being exposed to the stressor. Therefore, the repeat test takers are not unaccustomed to being more emotionally exhausted than the freshers. As aforementioned, burnout has a complete cycle, whereas emotional exhaustion is the first step, is followed by cynicism and decreased academic efficacy [1, 4]. Consistent with the cycle, the repeat test-taking students of the present sample might be reported with higher scores of the first step, and significant relationships between the two groups are observed herein.

For the betterment of life, there is no alternative to education. An academic achievement like getting admitted into a university is undoubtedly regarded as a societal success in Bangladesh [16]. Thus, there is an untold appetite among the eligible high school graduates appearing for the university admission tests. Besides, other academic achievements such as previous GPA, grades, exams, etc., are the potential factors of burnout [see Madigan and Curran [31] for a recent review on burnout and academic achievement], which is also reported in the present sample. For instance, participants with a higher or lower GPA compared to moderate ones have noted a higher burnout tendency along with comorbid symptoms like depression, anxiety, etc. [28]. Besides, satisfaction with the prior mock tests is also reported as the associated factor of burnout in this study. The obvious reasons explaining these unusual findings (i.e., the relationship between higher GPA and previous mock test satisfaction with burnout) are unpredictable to the authors’ best knowledge. However, further research can be carried out focusing academic satisfaction as a burnout causality with more rigorous methodologies.

There is a huge literature on negotiating relationship between burnout and mental health problems [1, 32]. For example, empirical investigation is carried out to establish the overlapping relationship between burnout and depression [9]. As a result, burnout is suggested to be a depression clinical picture by sharing similar signs and symptoms [i.e., loss of interest or pleasure, depressed mood, fatigue or loss of energy, impaired concentration, feelings of worthlessness, etc. [33]]. Thus, these two psychological conditions increase and decrease in a commensurate manner over time; the observation is supported by a longitudinal study among 3,255 Finnish healthcare professionals [34]. A consistent finding is reported in Thai medical students [29], whereas both depression and anxiety in higher burnout syndrome are found in Nepalese medical students [28]. For this reason, that anxiety is a psychological and physiologic state characterized by cognitive, somatic, emotional, and behavioral components [35], whereas prolonged anxiety raises the situation of individuals’ less productivity and functioning by mediating psychological distresses [36]. Likewise, Ding et al. [10] report that emotional exhaustion and cynicism are positively related to anxiety symptoms, whereas professional efficacy is negatively correlated. That is, the more emotionally exhausted, cynical, and less efficient toward their work an individual feels, the more anxious they will be. However, possibly participants diagnosed with a depressive and/or an anxiety disorder may also suffer from burnout suffering [9]. Consistent with the previous findings, the present study reports the common psychiatric suffering to be highly associated with burnout across three groups. That means participants suffering from depression and anxiety have a greater chance to develop burnout compared to those who are not.

## 5 Strengths and limitations

The present study can be limited because of its nature (i.e., cross-sectional study) and not including the test-taking students residing outside the university. Thus, further studies require replicating the objectives with more robust methodological designs (i.e., longitudinal or qualitative studies) and more representative samples. However, despite these limitations, the present study provides benchmark data on the sample concerning burnout for the first time.

## 6 Conclusions

The findings of this study warrant that the repeated test-taking students are at higher risk of developing burnout, where psychopathological factors also act as a significant contributing probable factor. As an initial assessment, the findings are anticipated to be helpful for policymaking and further studies inside and outside Bangladesh, whereas the university entrance test is mandatorily taken.

## Supporting information

S1 Dataset (SAV)
